# Systematic changes in circumpolar dust transport to the Subantarctic Pacific Ocean over the last two glacial cycles

**DOI:** 10.1073/pnas.2206085119

**Published:** 2022-11-21

**Authors:** Torben Struve, Jack Longman, Martin Zander, Frank Lamy, Gisela Winckler, Katharina Pahnke

**Affiliations:** ^a^Marine Isotope Geochemistry, Institute for Chemistry and Biology of the Marine Environment (ICBM), University of Oldenburg, 26129 Oldenburg, Germany;; ^b^Department of Marine Geology, Alfred Wegener Institute, Helmholtz Centre for Polar and Marine Research, 27568 Bremerhaven, Germany;; ^c^Lamont-Doherty Earth Observatory of Columbia University, Palisades, NY 10964;; ^d^Department of Earth and Environmental Sciences, Columbia University, NY 10027

**Keywords:** Southern Ocean, dust provenance, Southern Hemisphere westerly winds, subtropical jet, iron fluxes

## Abstract

The input of the soluble micronutrients iron (Fe) and/or manganese (Mn) by mineral dust stimulates net primary productivity in the Fe/Mn-deficient Southern Ocean. This mechanism is thought to increase carbon export, thus reducing atmospheric CO_2_ during the Pleistocene glacial cycles. Yet, relatively little is known about changes in the sources and transport pathways of Southern Hemisphere dust over glacial cycles. Here, we use the geochemical fingerprint of the dust fraction in marine sediments and multiisotope mixture modeling to identify changes in dust transport to the South Pacific Subantarctic Zone (SAZ). Our data show that dust from South America dominated the South Pacific SAZ during most of the last 260,000 a with maximum contributions of up to ∼70% in the early part of the glacial cycles. The enhanced dust-Fe fluxes of the latter parts of the glacial cycles show increased contributions from Australia and New Zealand, but South American dust remains the dominant component. The systematic changes in dust provenance correspond with grain size variations, consistent with the circumpolar transport of dust by the westerly winds. Maximum contributions of dust from more proximal sources in Australia and New Zealand (up to ∼63%) paired with a finer dust grain size indicate reduced westerly wind speeds over the South Pacific SAZ during deglacial and peak interglacial intervals. These quantitative dust provenance changes provide source-specific dust-Fe fluxes in the South Pacific SAZ and show how their systematic changes in magnitude and timing influence the Southern Ocean dust-Fe feedback on glacial-interglacial to millennial time scales.

## Significance

The deposition of mineral dust can stimulate primary productivity in the remote Southern Ocean through the supply of the micronutrient iron (Fe), leading to a removal of atmospheric CO_2_ especially during glacial periods. By analyzing the geochemistry of marine sediments, we show that dust-borne Fe input in the Subantarctic South Pacific was related primarily to the circumpolar transport of dust from South American sources. Increasing input from more proximal sources in Australia and New Zealand represents important secondary components during the latter part of the two glacial cycles. Our quantitative dust provenance data vary systematically with dust grain size and dust-Fe fluxes, highlighting how source-specific changes in Southern Hemisphere dust transport influenced the efficiency of Southern Ocean CO_2_ removal.

Atmospheric dust is an important component of the climate system, influencing the Earth’s radiation budget (1–3) and delivering (micro)nutrients to remote marine (4) and terrestrial ecosystems (5). The Southern Hemisphere dust cycle is of particular interest due to the large reservoir of unused macronutrients and the deficiency of the micronutrients iron (Fe) and manganese (Mn) in the Southern Ocean (6–9). It was hypothesized that the enhanced airborne supply of soluble Fe would reduce this micronutrient deficiency, thus stimulating net primary productivity and carbon export leading to a net drawdown of atmospheric CO_2_ (6, 10). However, results of experimental studies aiming to test the effect of Fe fertilization in the Southern Ocean were equivocal due to the temporal and spatial limitations of artificial and natural Fe fertilization experiments (7, 8, 11).

The marine sedimentary record provides evidence for large-scale natural Fe fertilization experiments during the late Pleistocene glacial-interglacial cycles (12, 13). Previous work from the South Atlantic demonstrated that increased dust-Fe input led to enhanced nutrient consumption and export productivity (i.e., the net export of organic carbon into the seafloor sediments) in the South Atlantic Subantarctic Zone (SAZ) during past glacial intervals (12–14). These findings are consistent with reconstructions of dust input from the South Pacific SAZ (15, 16), confirming the linkages between Southern Ocean SAZ dust-Fe input, primary productivity, atmospheric CO_2_ concentrations, and temperature on glacial-interglacial timescales (12, 13, 15–17). In contrast, the South Atlantic Antarctic Zone (AZ) shows the opposite pattern with declining export productivity during glacial intervals of high dust input, thus precluding a pronounced role for this area in glacial dust-Fe fertilization (17). Overall, the dust-climate feedbacks were suggested to account for a net drawdown of atmospheric CO_2_ of up to ∼40 ppm during past glacial periods, i.e., almost half of the total glacial-interglacial change (3, 13, 17, 18).

**Fig. 1.**
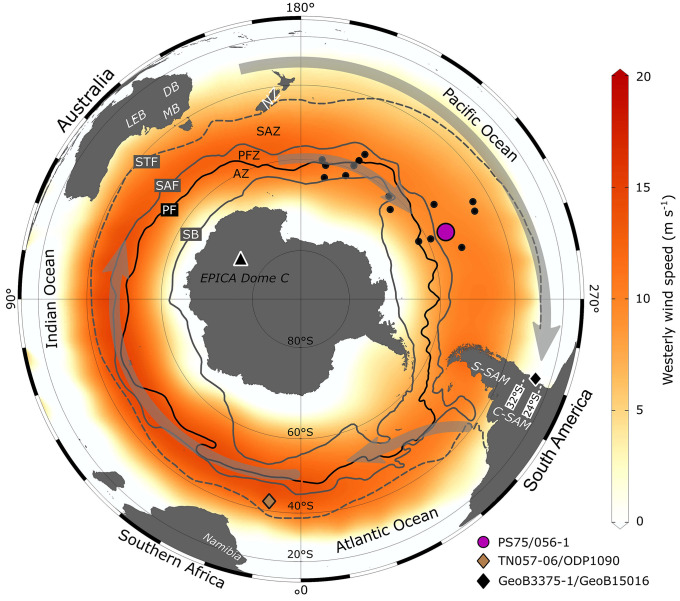
Polar map of the Southern Hemisphere. Annual means of NCEP/NCAR westerly wind speeds at 850-hPa geopotential height (red shading, in m s^−1^) for the period 1948 to 2021 (102). Gray arrows indicate the midlatitude jetstream circulation at 200 hPa (69). Note the (winter-time) split jet in the South Pacific (polar and subtropical branch). Nonwesterly winds appear as white areas. Important subcontinental-scale PSAs are indicated in white italics. LEB, Lake Eyre Basin; DB, Darling Basin; MB, Murray Basin; NZ, New Zealand. South American dust PSAs are grouped by latitude. Key locations included in the discussion are as follows: Antarctic ice core EPICA Dome C (68, 103), Atlantic Ocean cores TN057-06 (16) and ODP1090 (12, 13), South Pacific core PS75/056-1 (15, 16, 48), Chile margin cores GeoB3375-1 and GeoB15016 (41), and South Pacific LGM dust provenance time slice cores (black dots) (51, 52). STF, Subtropical Front; SAF, Subantarctic Front; PF, Polar Front; SB, Southern Boundary of the Antarctic Circumpolar Current; PFZ, Polar Frontal Zone. AZs/fronts are from ref. 104. Base map was produced with Ocean Data View software (105). S-SAM, Southern South America; C-SAM, Central South America; NCEP, National Centers for Environmental Prediction; NCAR, National Center for Atmospheric Research.

The emission of dust requires specific conditions in the potential dust source areas (PSAs). They include a low soil moisture content (at least during parts of the year), the (re)supply of fine material for deflation, and low vegetation cover for efficient dust entrainment (19–21). These key conditions are present in the (semi)arid continental interior regions of Australia, South America, and South Africa that are typically considered as the three major dust sources in the Southern Hemisphere (19, 21–24). The resupply of fine particles in the Southern Hemisphere PSAs is linked predominantly to fluvial and lacustrine processes in (ephemeral) river plains and lake basins of the (semi) arid continental interiors (19, 21). In addition, glacial and glacio-fluvial processes erode large amounts of sediments in the Southern Alps in New Zealand (25, 26) and the South American Andes (27–30). On the relatively dry leeward side of the mountain ranges (rain shadow), the fine fraction of these glaciogenic deposits can be entrained by winds, especially during glacial intervals of increased glacier activity and when a lower sea level exposed large shelf areas susceptible to eolian deflation (26, 31–36). The moisture supply for these processes in the source regions is largely regulated by the tropical monsoon circulation and/or the midlatitude Southern Hemisphere westerly winds (SWWs) with pronounced seasonal to millennial-scale variations (20, 29, 30, 37–41). The long-distance transport of dust from the continental source regions across the midlatitude Southern Hemisphere oceans is, however, dominated by the westerly winds (19, 23, 24, 42) (Fig. 1).

Past changes in the provenance and transport routes of far-traveled dust in the extratropical Southern Hemisphere are primarily derived from the geochemical fingerprint of dust particles in Antarctic ice cores compared to PSA signatures (22, 26, 43–47) and modeling studies (23, 24, 42), suggesting that Patagonia is the dominant source of far-traveled dust to East Antarctica during glacial periods (22, 33, 34, 43–46). However, Antarctic ice core data provide only indirect evidence on dust provenance and transport in the key region of glacial dust-Fe fertilization, the Southern Ocean SAZ.

Marine sediment records from both the South Atlantic (12) and South Pacific SAZ (15, 16) resemble the pattern of glacial-interglacial variability of Antarctic dust deposition. However, the particle size of the dust fraction shows contrasting behavior over the last two glacial cycles between the Atlantic and the Pacific SAZ (48). As airborne dust particle size is controlled primarily by gravitational settling (2, 49), these differences were suggested to result from changes in dust sources, namely, wind and transport patterns specific to the different sectors of the Southern Ocean (48). Recent provenance studies support a more nuanced picture of dust sources and transport routes in the Southern Hemisphere during glacial and interglacial periods (36, 50–52).

Importantly, variations in source area mineralogy (provenance), transport pathways, and travel times of atmospheric mineral dust all have critical influence on the solubility and bioavailability of dust-borne Fe (16, 53–59). However, the exact links between dust provenance, transport, and input fluxes in the remote South Pacific SAZ are still unclear for full glacial-interglacial cycles. Therefore, identifying the sources and travel routes of dust input to the South Pacific SAZ over full glacial cycles is instrumental to understand the dust-climate feedbacks associated with the Southern Hemisphere dust cycle. Here, we use a set of complementary but independent geochemical tracers including rare earth elements (REEs), strontium (Sr), neodymium (Nd), and lead (Pb) isotopes from marine sediment core PS75/056-1 to constrain sources and transport paths of dust delivered to the South Pacific SAZ over the last 260,000 y. We quantify the individual PSA contributions to the total Fe flux (15, 16) at the core location by using an isotope mixing model. In combination with existing grain size data from core PS75/056-1, the quantification of dust provenance changes provides insight into possible changes in SWW intensity in the Pacific sector of the Southern Ocean.

## Results and Discussion

### Geochemical Fingerprint and Source Apportionment of Subantarctic South Pacific Dust.

**Fig. 2.**
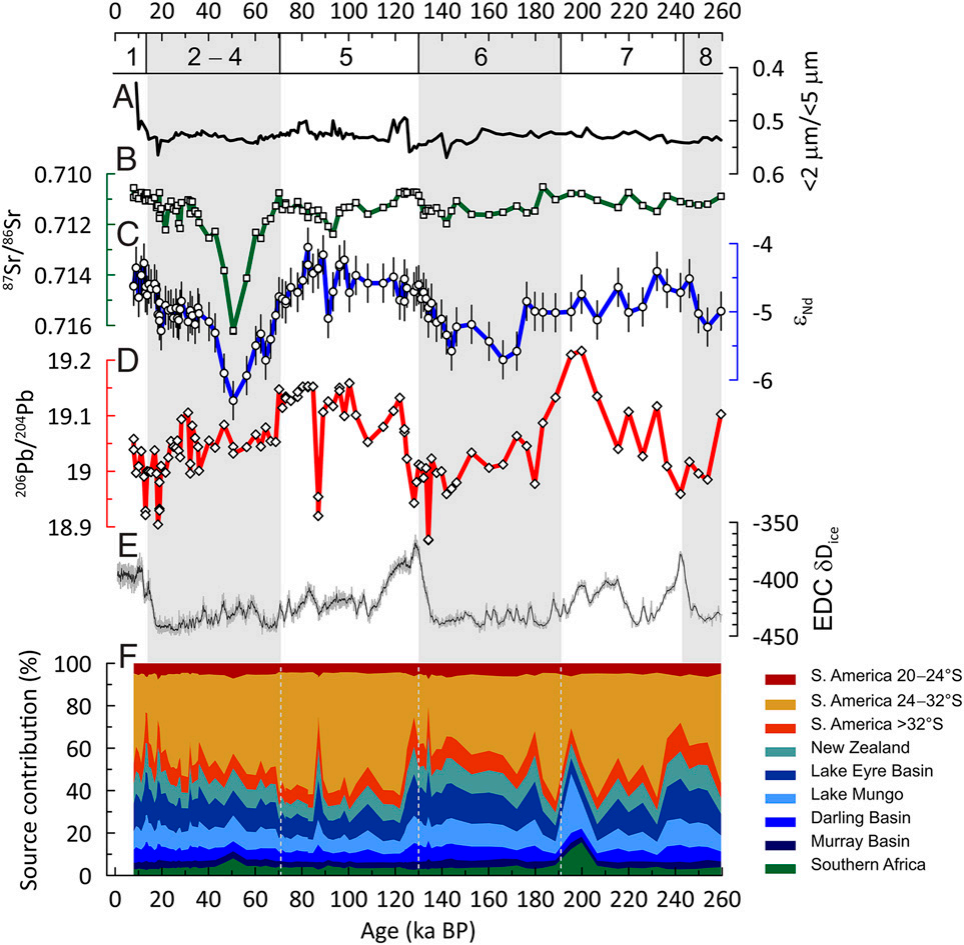
Geochemical provenance data and source apportionment for core PS75/056-1. (*A*) Proportion of <2-µm fraction (containing clay minerals) in the <5-µm fraction investigated in this study. (*B*) Sr isotope results presented as ratio of ^87^Sr/^86^Sr. (*C*) Nd isotope data expressed in epsilon notation (*Materials and Methods* contains more details). (*D*) Pb isotope results with ^206^Pb/^204^Pb ratio depicted. Error bars are shown where calculated 2SD uncertainty is larger than the symbol size (*Materials and Methods* contains more details). (*E*) EPICA Dome C ice core deuterium data superimposed as stratigraphic reference (103). (*F*) Source apportionment based on the Bayesian mixing model MixSIAR and available terrestrial dust source data (*Materials and Methods* and *SI Appendix* contain more details). MISs are indicated by numbers below the upper x-axis, which are gray bars and gray stippled lines in (*F*).

The dust fraction isotope data show pronounced changes over the last ∼260,000 y (Fig. 2). The Nd isotope compositions range from ε_Nd_ = −4.1 ± 0.3 to −6.3 ± 0.3 and Sr isotope compositions from ^87^Sr/^86^Sr = 0.71051 ± 0.00003 to 0.71623 ± 0.00003 (Fig. 2 *B* and *C* and Dataset S1). Both isotope systems show a distinct shift during Marine Isotope Stage (MIS) 4 and MIS 3 (between ∼70,000 and 40,000 a before present [BP, present = 1950 CE]) from a background with relatively small variation throughout the past 260,000 y (Fig. 2 *B* and *C*). The Pb isotope results for ^206^Pb/^204^Pb range from 18.876 ± 0.004 to 19.217 ± 0.004 with the most radiogenic Pb isotope compositions during MIS 2, 5a-d, 7, and 8 (Fig. 2*D* and Dataset S1). Our complementary REE data exclude the influence of authigenic seawater-derived and/or hydrothermal phases in the extracted signal, and the flat shale-normalized REE patterns suggest a crustal source for our samples (*SI Appendix*, Fig. S2). This crustal signal demonstrates the insignificant influence of volcanic ashes on the source composition, which would show pronounced, simultaneous, and erratic shifts of all our provenance tracers (60). This is not evident in our downcore dataset (Fig. 2 *B*–*D* and *SI Appendix*, Fig. S2).

Notably, our downcore data show that the relative abundance of clay-sized particles (<2 µm) is not a first-order control on the Sr isotope composition of the <5-µm fraction (Fig. 2*A*) reflected by a Pearson correlation coefficient of *r* = 0.14 and a *P* value of 0.17 (*n* = 100). This is important because the Sr isotope composition is particularly sensitive to grain size and/or mineral sorting processes, as the formation of clay minerals from weathering fluids can lead to the enrichment of radiogenic ^87^Sr in the sediment fine fraction (61, 62), typically referred to as the grain size effect (63). Nevertheless, size fractionation during emission, transport, and/or deposition can lead to a bias between the dust composition at the source and the composition at the site of deposition (63, 64). For this reason, previous dust provenance work in the South Pacific focused on tracers that are typically less sensitive to grain size effects, e.g., Nd and Pb isotopes (51). Recent tests with different mixing model configurations demonstrate that source apportionment in the dust fraction of South Pacific marine sediments shows only modest changes between setups with and without Sr isotopes (52). However, our data show a considerable ^87^Sr/^86^Sr change of ∼0.005 coinciding with an ε_Nd_ excursion of ∼2 between ∼70,000 and 40,000 y BP (Fig. 2 *B* and *C*). As we can exclude a primary control by clay mineral abundance (Fig. 2*A*), we ascribe this distinct feature to provenance changes and included the Sr isotope system in the dust provenance modeling.

Our dataset also shows short-lived Pb isotope shifts toward unradiogenic compositions (e.g., at ∼87,000 and 134,000 y BP) (Fig. 2*D*), which may indicate an influence from grain size and/or mineral sorting effects on the Pb isotope composition of the dust fraction samples from core PS75/056-1. However, the influence of sorting effects on the Pb isotope composition of Southern Hemisphere PSA (33, 65) and far-traveled dust in the South Pacific is typically low (51). Moreover, there is no systematic correspondence between the Pb isotope shifts with complementary Sr isotope and grain size data that would support a role for sorting effects (Figs. 2 *A*, *B*, and *D* and 3*B*). Therefore, we consider the excursions to reflect short-lived changes in dust provenance (which may imply mineralogical changes), generally consistent with the nature of dust storms observed in the main Southern Hemisphere PSAs (66, 67) and with high-resolution geological records showing spike-like changes in dust fluxes (12, 15, 16, 68).

Overall, the ranges of Pb, Sr, and Nd isotope compositions in our PS75/056-1 downcore dataset are similar to the range of dust signals in the polar and subpolar South Pacific during the Last Glacial Maximum (LGM) (*SI Appendix*, Figs. S3–S5) (51). Accordingly, the model simulations tally with recent source apportionment for these LGM samples (52). In agreement with previous findings (51, 52), the downcore variability in dust provenance is dominated by Pb isotope changes (Fig. 2 *D* and *F*). This can be ascribed to the small change in sample Nd and Sr isotope compositions relative to the compositional ranges of the dust source areas in the Southern Hemisphere (*SI Appendix*, Table S2).

**Fig. 3.**
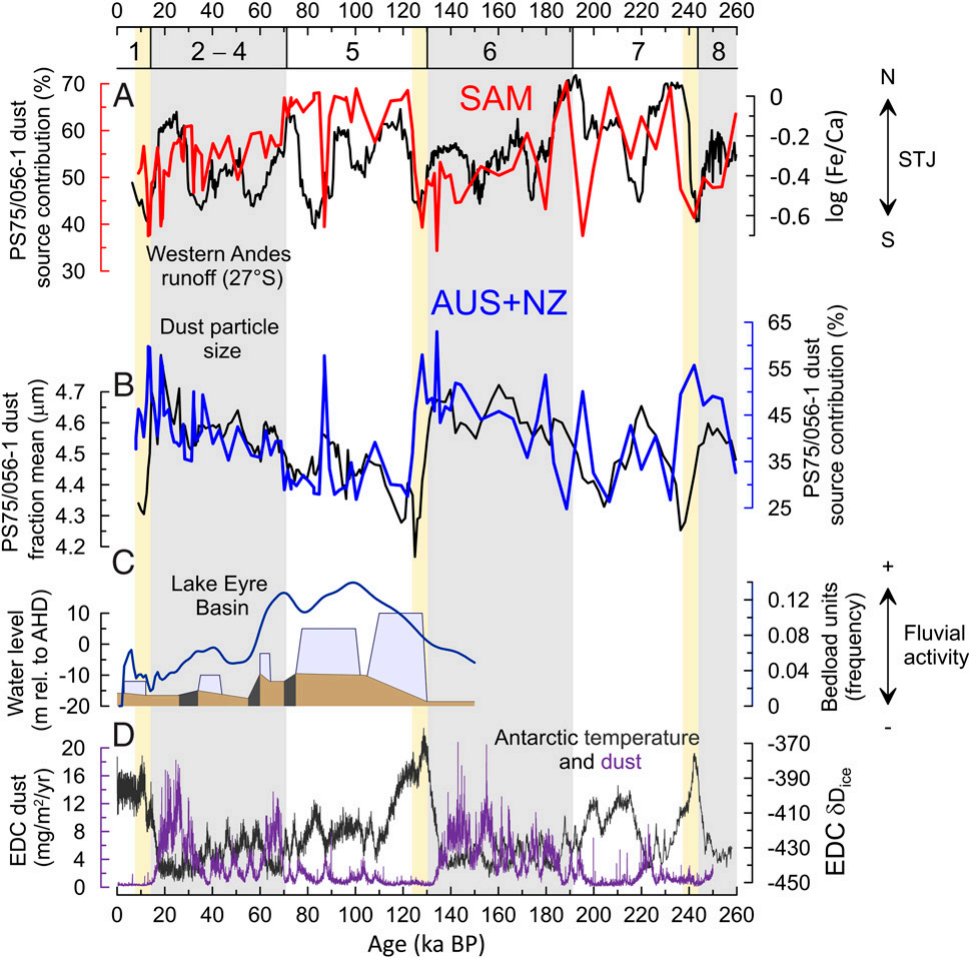
Grouped dust source contributions and indicators of dust transport dynamics. (*A*) South American (SAM) contribution to the total deposition in core PS75/056-1 compared with fluvial runoff from the western Andes reconstructed from Fe/Ca ratios in cores GeoB3375-1 and GeoB15016 as an indicator for the presence of the STJ at ∼27°S (41) (see also Fig. 1). (*B*) Australian/New Zealand (AUS + NZ) contribution to the total deposition in core PS75/056-1 compared with the mean of the 2- to 10-µm fraction considered to represent dust input at location PS75/056-1 (48). (*C*) Lake Eyre water level in pale blue and lakebed in brown (in meters relative to AHD [Australian Height Datum]) with dark-gray bars indicating lakebed erosion/deflation (73, 75). Frequency of bedload units (1-ka bins) indicate fluvial activity in the Lake Eyre Basin (74). (*D*) Antarctic EPICA Dome C (EDC) deuterium data as temperature indicator (103) with EDC dust flux record superimposed (68). MISs are indicated by numbers below upper x-axis and gray bars. Yellow bars indicate deglacial and peak glacial intervals with decreasing grain size and elevated AUS contribution that are indicative of reduced wind speed in the Subantarctic South Pacific (see text for more details).

The source apportionment shows that two continental-scale source regions dominated the dust input in PS75/056-1 over the last 260,000 y, i.e., Australia/New Zealand (∼25 to 63%, average of ∼42%) and South America (∼34 to 70%, average of ∼55%). Southern Africa plays a minor role (3 to 16%, average of ∼4%) (Fig. 2*F* and Dataset S2), except during the intervals from ∼40,000 to 70,000 y BP and from ∼190,000 to 200,000 y BP (Fig. 2*F*). On a subcontinental scale, South American PSAs between 24 and 32°S (Southern Puna and parts of northwestern Argentina) dominate the total dust deposition in PS75/056-1 with contributions of up to ∼60% (Fig. 2*F*). The highest fractions of dust from South American sources are found in the early part of the last two glacial cycles followed by increasing contributions from Australia/New Zealand during the later parts. However, South American sources remained the dominant dust supplier at PS75/056-1 over the last 260,000 y, except for deglacial and peak interglacial intervals when the majority of the dust delivered to the South Pacific SAZ originated from Australia/New Zealand (Fig. 3*B*).

### Dust Provenance and Transport Dynamics in the Subantarctic South Pacific.

The source apportionment shows that Australian/New Zealand and South American PSAs explain typically ≥95% of the dust deposition in core PS75/056-1 with systematic changes over the last ∼260,000 y (Dataset S2 and Fig. 2*F*), so we focus our discussion on the changing contributions from sources on these two continents.

Central South America stands out as the single most important subcontinental scale PSA contributing up to ∼65% to the total dust deposition at our core location during the last 260,000 y when also including the small contribution from the Northern Puna and Altiplano Plateau between 20 and 24°S (average of ∼5%) (Figs. 1 and 2*F*). This result is different to the general view that Patagonia is the most dominant source region for far-traveled dust from South America (22, 27, 43). Yet, it is generally consistent with earlier work that considered the high elevation, high relief regions in Central South America as active emitters of far-traveled dust today and in the past (33, 34, 45, 64, 67), likely supported by recurring glacier advances increasing sediment availability at times (28–30). Dust transport from these high elevation source regions in Central South America has been linked to the intersecting subtropical westerly jet (STJ) circulation (64, 67). At present-day, the STJ is centered at around ∼30°S in the South Pacific during austral winter (69) (Fig. 1) and corresponds with an equatorward expansion of the SWW in the lower troposphere, leading to a pronounced rain shadow on the dry eastern flank of the Central Andes (39). Therefore, the STJ has been proposed to play an important role in the transport of dust from the high-altitude source regions on the eastern flank of the Central Andes into the South Pacific on a circumpolar trajectory during Holocene and LGM times (51, 52). Observations from the Northern Hemisphere provide an example of how the dust plumes travel around the globe with the jetstream (70). Once at high tropospheric levels, the dust plume traveled rapidly eastward with sporadic dust deposition during its circumpolar journey (70). The authors also identified that large-scale subsidence of air masses in high-pressure systems promotes the deposition of dust from high tropospheric levels (70). Accordingly, we suggest that dust emitted at high altitudes in Central South America is transported by the STJ (Fig. 1). While dust removal would occur throughout the whole circumpolar journey, enhanced deposition of dust particles is expected in the large-scale high-pressure systems in particular in the South Pacific.

Reconstructions show that the strength and/or position of the westerly wind system changed on orbital to (sub)millennial time scales (41, 71–73). Our downcore data show qualitative agreement between elevated South American source contributions and stronger STJ presence at ∼27°S off western Chile (41), especially for the last glacial cycle (MIS 1 to 5) where our dust provenance record provides the highest resolution (Fig. 3*A*). However, we also observe significant differences throughout the investigated time interval. These differences may (partly) be related to age model uncertainties of several thousand years between the two records (41, 48) or to changing environmental conditions in one of the source areas unrelated to the direct interaction of Central South American dust emissions with STJ dynamics. Environmental changes influencing the production of fine particles and dust activity in the Southern Hemisphere PSA can be driven by tropical circulation cells and moisture source changes modulating hydrological and glacial activity in the main dust source regions of Australia (74–76) and Central South America (28–30). For example, our data show that the highest South American contributions—up to ∼70%—occur early in the two glacial cycles when the total dust fluxes are relatively low in the Southern Hemisphere (12, 15, 16, 68, 73) (Fig. 3 *A*, *B*, and *D*). Particularly high contributions from South American sources coincide with high lake levels and river activity in the Lake Eyre Basin during MIS 5 (74–76) (Fig. 3*C*). This suggests that increased moisture fluxes related to changes in the Australasian ITCZ/monsoon circulation (74, 75) could have reduced dust emissions from Australian sources during these periods. We note that seasonal increases in fluvial activity can enhance sediment availability on river plains and promote dust generation (21, 37).

The contributions of dust from subcontinental-scale PSAs in Australia and New Zealand changed rather systematically and synchronized over the last ∼260,000 y, typically in an opposite direction to the proportion of Central South American dust in PS75/056-1 (Figs. 2*F* and 3 *A* and *B*). This can be expected due to a dilution effect in a binary mixing system, but considering the different environmental conditions and geochemical fingerprints of Australian and New Zealand PSAs (26, 37, 47, 74, 77), their synchronous behavior is more likely related to a shared transport mechanism that is different to the dust transport from Central South America. The dust source regions in Australia and New Zealand are located primarily at relatively low elevations (35, 47). As dust is subject to rapid wet removal within the lower troposphere SWW (49, 73, 78), the long-distance transport of dust from Australian/New Zealand PSAs is dependent on uplift to higher tropospheric levels (35, 37, 38, 47, 66). This is in contrast to dust emissions from high-elevation sources in Central South America, which can be entrained directly at high altitudes into the STJ (67). Therefore, we propose that the dust provenance variability recorded in PS75/056-1 results from sediment availability in the source regions and the interplay of STJ-transported dust from high elevation sources in Central South America and dust emitted from PSAs at lower atmospheric levels in Australia/New Zealand with faster (wet) removal from the atmosphere within the frontal systems of the SWW (49, 73, 78).

**Fig. 4.**
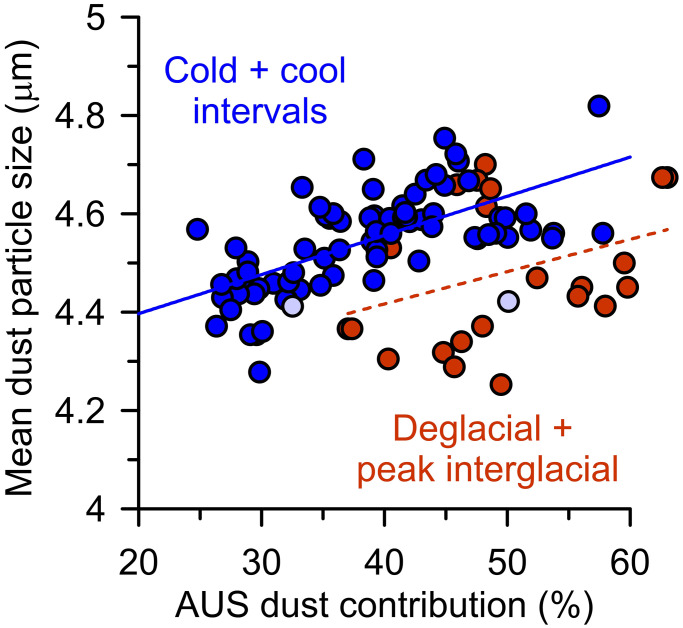
Relationship between dust particle size and provenance. For cold and cool intervals (including MIS 2 to 4, 5a-d, 6, late MIS 7, and 8), Pearson’s r yields 0.65 (*n* = 74, *P* <0.01, two samples with high contributions from Southern Africa indicated by symbols with pale-blue filling). For deglacial and peak interglacial samples, Pearson’s r yields 0.34 (*n* = 26, *P* = 0.09). Dataset S2 contains the exact categorization of individual samples. Note that the interpolation of the Australian/New Zealand (AUS) dust contribution to 100% (= 0% from South America) yields a mean dust particle size of ∼5.04 µm and interpolation of the South American contribution to 100% gives ∼4.19 µm for cold and cool intervals.

This is further corroborated by the striking correspondence between dust provenance and grain size in core PS75/056-1 (Fig. 3*B*). Higher contributions from South American sources typically correspond with finer dust grain sizes after peak interglacial conditions, followed by an increased proportion of Australian dust and a coarsening of the dust grain size in core PS75/056-1 during the latter part of the glacial cycles (Fig. 3 *A* and *B*). This relationship between the proportion of Australian dust and grain size is expressed in a Pearson correlation coefficient r of 0.65 for “cold and cool” intervals in core PS75/056-1 (*n* = 74, *P* < 0.01; both parameters from the same samples, excluding two samples where South African contributions increase above 10%) (Fig. 4). This correlation also suggests that the variations in dust grain size (48) are largely controlled by dust provenance in the South Pacific SAZ during these intervals. The provenance of dust can influence the grain size of far-traveled dust by changes in the particle size distribution at emission in the different source regions and/or by different travel times via the preferential removal of coarser dust particles during airborne transport (49, 78). The particle size distribution at emission is rather similar for the <5-µm dust fraction across different analytical methods and soils (49, 79). Also considering varying environmental conditions during the long phases categorized here as cold and cool (category defined in caption of Fig. 4 and indicated in Dataset S2) (Figs. 3 *A*, *C*, and *D* and 4), changes in particle size distribution at emission are unlikely to be the dominant control on the consistent and systematic relationship between dust provenance and grain size in PS75/056-1 during these intervals. Therefore, we suggest that the provenance-related dust grain size changes in PS75/056-1 are driven primarily by the preferential removal of coarser dust particles during transport (49, 78), i.e., the longer circumpolar journey of South American dust leads to increased removal of coarser particles in comparison to dust from more proximal Australian/New Zealand sources (Fig. 3*B*).

Interestingly, previous work identified the relative increase of a non-Patagonian dust component in East Antarctic ice core samples from EPICA Dome C (Fig. 1) during glacial intervals of high total dust fluxes in the Southern Hemisphere (45). We note that a comprehensive and quantitative comparison with our dataset is limited by the large analytical uncertainties of currently available Pb isotope data resulting from the low Pb concentrations in Antarctic ice core samples (46). However, the relative increase of Australian/New Zealand dust during peak glacial conditions in core PS75/056-1 suggests that these sources contributed to the increase of non-Patagonian dust in East Antarctica during glacial intervals of the Pleistocene (45). This implies that Australian/New Zealand dust would have influenced large parts of the Southern Hemisphere at these times. Deviations from the systematic glacial-style relationship between dust grain size and provenance in core PS75/056-1 are evident for the glacial terminations 1 to 3 and the following peak interglacial conditions of MIS 1, 5, and 7 (Fig. 3*B* and *D*). During these periods, high contributions from Australian and New Zealand sources of up to ∼60% are associated with decreases in the dust grain size. The grain size of far-traveled airborne dust depends on the wind speed during transport and the travel time and distance (49, 78, 79). Therefore, the high contribution of dust from more proximal Australian/New Zealand sources paired with reduced dust grain sizes implies a reduction in wind strength over the South Pacific SAZ (Fig. 1). A reduction in westerly wind strength near Australia is consistent with a more poleward position of the SWW core during these intervals (71). Our results demonstrate that dust source apportionment allows unequivocal interpretation of grain size data with regard to changes in wind strength in the South Pacific SAZ and provides important constraints on the atmospheric dust transport pathways in the Southern Hemisphere over Pleistocene glacial cycles.

### Source-Specific Fe Fluxes to the Subantarctic South Pacific.

**Fig. 5.**
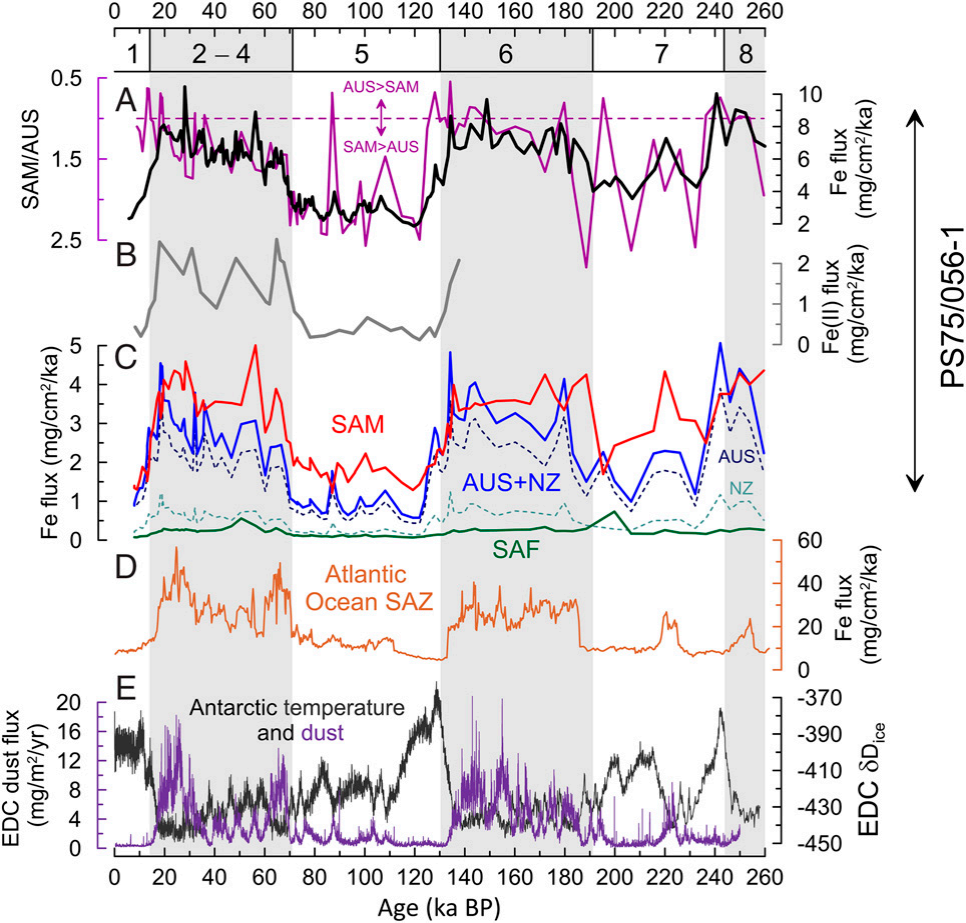
Dust-induced iron input to the Subantarctic Southern Ocean. (*A*) Ratio of relative dust contributions from South America (SAM) and Australia/New Zealand (AUS) in purple compared to Fe fluxes for core PS75/056-1 (16). (*B*) Fe(II) fluxes for PS75/056-1 (16). (*C*) Fe fluxes for PS75/056-1 subdivided according to dust provenance apportionment for SAM (red solid line), AUS (dark-blue stippled line), Southern Africa (SAF, green solid line), New Zealand (NZ, ocean green stippled line), and Australia with New Zealand combined (blue solid line). (*D*) Fe fluxes for the South Atlantic SAZ from core ODP 1090 (light brown) (12). (*E*) EDC deuterium data as temperature indicator (103) with EDC dust flux record superimposed (68). MISs are as in Fig. 3.

The grouped continental-scale dust provenance variability shows systematic correspondence with Fe flux changes in core PS75/056-1 (Fig. 5*A*). Low dust-Fe fluxes are supported primarily by South American input, whereas high dust-Fe fluxes correspond with increasing contributions from Australian/New Zealand sources. The relationship between dust provenance changes and Fe fluxes (Fig. 5*A*) is reflected in a Pearson correlation coefficient of 0.72 (*P* < 0.01, *n* = 79) for the cold intervals and a weaker, but statistically still significant correlation of 0.59 (*P* < 0.01, *n* = 26) for deglacial and peak interglacial periods (grouped as in Fig. 4). This demonstrates that dust provenance changes were an inherent feature of the Southern Hemisphere dust-Fe feedback over the last two glacial cycles, both during intervals of increasing and decreasing Fe input to the South Pacific SAZ.

Using previously published data from PS75/056-1 (15, 16), we convert the relative dust source contributions in PS75/056-1 into absolute dust-Fe fluxes from the main continental source regions (Fig. 5*C*). The resulting pattern of calculated dust-Fe fluxes for PS75/056-1 shows higher fluxes from both Australia/New Zealand and South America during glacial times (MIS 2 to 4, 6, and 8) as opposed to interglacial times (MIS 1, 5, and 7). The overall glacial-interglacial patterns of dust-Fe contributions to PS75/056-1 from both continents resemble the pattern of dust-Fe fluxes in the South Atlantic SAZ (12, 73) and Antarctica (68) (Fig. 5 *D* and *E*). Generally higher total dust-Fe fluxes in the South Atlantic SAZ are consistent with a reduced distance to the respective dust source regions in comparison to site PS75/056, which is located at a maximum distance from all possible dust source regions under SWW/STJ influence (15, 16) (Fig. 1). These observations seem to support the idea of a common large-scale climate forcing driving the glacial-interglacial changes of dust input from different sources in the individual sectors of the Southern Ocean SAZ (15, 16) and globally (80).

The interglacial-glacial rise in dust-Fe flux is greater for Australian input (∼fourfold to fivefold increase) than that for South America (∼threefold increase) (Fig. 5*C*). Despite this increase of total Fe fluxes from Australia, the continent is typically not considered a significant source of glaciogenic sediments (16, 47, 77). In contrast, New Zealand has previously been suggested as a source of both increased total Fe (15) and Fe(II)-rich silicate mineral fluxes during glacial intervals (16). In particular, the similar phasing and proportion of primary Fe(II)-rich minerals in glacial sediments from PS75/056-1 and South Atlantic core TN057-06 have been ascribed to the synchronous emissions of glaciogenic dust from Patagonia and New Zealand (15). Our provenance apportionment shows that the fraction of dust from New Zealand increases by a factor of ∼2 in PS75/056-1 during glacial intervals as compared to the interglacial background (Fig. 2*F* and Dataset S2), consistent with a substantial increase in glacial coverage and shelf exposure supporting enhanced dust emissions at these times (26). This higher proportion of dust from New Zealand translates into an ∼fourfold to sixfold increase in absolute Fe fluxes from New Zealand to the South Pacific SAZ during the MIS5/4 and MIS7/6 transitions, which are critical intervals of enhanced glacial dust flux and cooling via the Southern Ocean dust-Fe feedback (12, 13, 16) (Fig. 5*B*). However, the overall low dust-Fe fluxes from New Zealand (average of ∼0.7 mg/cm^2^/ka for MIS 2 to 4) are unable to explain Fe(II)-silicate fluxes of ∼2 mg/cm^2^/ka in PS75/056-1 during MIS 2 to 4 (Fig. 5 *B* and *C*). For southern South America (>32°S, incl. Patagonia), we obtain an average Fe flux of ∼0.6 mg/cm^2^/ka (i.e., ∼9% of total Fe flux at PS75/056-1 during MIS 2 to 4; Dataset S2). This implies that the two PSA previously suggested to drive the glacial increase in Fe(II)-silicate flux to the Southern Ocean SAZ (16) can account for a maximum of ∼65% of the total Fe(II)-silicate input—in the unlikely scenario that all dust-Fe from these sources was in Fe(II) form (57). Consequently, substantial contributions from other sources are required to explain the high Fe(II)-silicate fluxes in the South Pacific SAZ during glacial intervals. We suggest that Central South America played an important role for the glacial Fe(II)-silicate supply due to its prominent contribution to the total Fe flux (average of ∼3.1 mg/cm^2^/ka for MIS 2 to 4) (Fig. 2*E*).

Mineral dust originating from physical rock weathering in (sub)glacial environments carries higher proportions of reactive particulate and potentially bioavailable Fe in the form of primary Fe(II)-rich silicate minerals (e.g., biotite, hornblende), nanoparticles, and ferrihydrites (16, 57, 59, 81, 82). During cold stages, enhanced glacial activity in the high relief environments of the Central Andes (28) would have increased the production of reactive particulate Fe phases (16, 59, 81, 82). The predominating arid climate conditions in that region imply low chemical weathering rates supporting the preservation of primary Fe(II) silicate minerals (83, 84). Consequently, the dust-Fe emissions from multiple PSAs all contributed to different degrees to stimulate primary productivity (16, 57), nutrient consumption (13, 14), and export production in the Southern Ocean SAZ (12, 15, 73), thus supporting an increased sequestration of carbon in the deep ocean during glacial intervals (10, 18).

On millennial time scales, the timing of changes in source-specific dust-Fe fluxes can be different (Fig. 5 *A* and *C*). In particular, South American dust-Fe fluxes show a relatively gradual decline during the early deglacial intervals of terminations 1 and 2, which correspond with a weakening of the STJ off western Chile (*SI Appendix*, Fig. S6), facilitating the circumpolar transport of dust from South America into the South Pacific SAZ. In contrast, the deglacial structure of Australian dust-Fe flux changes shows rather step-wise reductions and intermittent increases during the early deglacial warming phases (*SI Appendix*, Fig. S6). This demonstrates how source-specific dust-Fe transport to the South Pacific SAZ could have acted as an early amplifier of deglacial climate amelioration through a reduction in the Southern Ocean dust-Fe feedback (3).

As such, the quantification of dust input from individual Southern Hemisphere PSAs to the South Pacific SAZ provides insight into their role for the Southern Ocean dust-Fe feedback from glacial-interglacial to millennial time scales over the last 260,000 y. A common large-scale climate forcing appears as an important control on circumpolar dust-Fe transport dynamics over glacial-interglacial time scales. However, PSA-specific responses to this large-scale climate forcing and millennial-scale variations suggest that the magnitude of dust-Fe flux variability from the individual Southern Hemisphere PSA is also influenced by the interaction of changes in environmental conditions in the source areas with the prevailing westerly wind system both on glacial-interglacial and millennial time scales. Implementing these results into future model designs will help to further refine the role of the Southern Ocean dust-Fe feedback during glacial-interglacial and millennial-scale climate change.

## Materials and Methods

For this study, we processed a total of 108 samples (including 13 full replicate samples; *SI Appendix*, Fig. S1) from sediment core PS75/056-1 (55.162°S, 114.789°W) in the South Pacific SAZ (Fig. 1). Sediment core PS75/056-1 was chosen because this core 1) has a robust age model largely based on benthic oxygen isotope stratigraphy (i.e., independent of dust input) (48), 2) is located in an area considered representative for the input of far-traveled dust to the South Pacific SAZ (48, 50, 51), and 3) provides a unique set of complementary dust input tracers including grain size analysis (48) and ^230^Th-normalized dust flux reconstructions (16). Our sample coverage yields an average time resolution of ∼3,000 y for the length of the core, which represents the time period between 8,000 and 260,000 y BP.

### Sample Processing.

The mineral dust signal has been extracted from the bulk sediment samples following previously published procedures (50, 51). In brief, the <63-µm fraction was separated from the bulk sample by wet sieving followed by Stokes-based gravimetric settling to obtain the <5-µm sediment fraction (51). This size fraction dominates the airborne lithogenic particle input in PS75/056-1 samples used for this study (15, 16, 48) and reduces possible bias from grain size effects (see also discussion above), thus allowing a better comparison with geochemical datasets from terrestrial PSAs and Antarctic ice core data (45, 64, 85). Moreover, the high cohesiveness of the <5-µm fraction minimizes resuspension and postdepositional transport by bottom currents (86). To avoid any possible bias of the dust fraction from biogenic and/or hydrogenetic sediment components, the <5-µm sediment fraction was subjected to a sequential leaching procedure, eliminating organics, carbonate, and ferromanganese (oxy-)hydroxides (51).

A subsample of typically an ∼50- to 60-mg freeze-dried <5-µm fraction sample material was fully digested using a mixture of concentrated HF-HNO_3_-HClO_4_ and the PicoTrace DAS pressure digestion system (51). The dissolved samples were converted to chloride form, and a split aliquot was taken for REE analyses. Following published protocols (50, 51), the remaining sample was converted to bromide form for an HBr-HNO_3_-based Pb extraction using Biorad AG1-X8 resin (87). The matrix wash fraction was collected to separate the alkaline earth metals (including Sr) from the REE using Biorad AG50W X-8 resin (88). Sr was separated from the remaining sample matrix using Eichrom Sr spec resin (89), and Nd was isolated from the LREE with Triskem Ln spec–based chemistry (90). A subset of samples was processed through a refined protocol collecting the Pb fraction from the Biorad AG1-X8 resin with 1.5 M HCl before eluting the Fe-containing sample matrix with 3 M HCl and collecting the remaining target fractions as described above (50, 88). The Sr fraction was then rejoined with the Pb fraction for separation from the remaining matrix elements using Eichrom Sr spec resin (modified after ref. 91). The purified Sr and Pb fractions were eluted sequentially with 0.05 M HNO_3_ and 6 M HCl, respectively (91).

### Trace Element Analyses.

Trace elements were analyzed on an aliquot of the fully digested sample in a 2% HNO_3_ matrix by using a ThermoFinnigan *Element II* inductively coupled plasma mass spectrometry (ICP-MS) instrument. Following the procedures outlined by ref. (51), the sample analyses comprised internal normalization by using a beryllium-indium solution and quantification of Ba, La, and Ce oxide formation at the beginning of each analytical session. Typical sample Ba/Eu ratios were ∼800 so that intereferences from Ba oxides (BaO+) exceeded the 2 relative SDs (2RSDs, in %) precision of our measurements. Therefore, we corrected all samples for BaO+ interferences on Eu by using a synthetic solution containing Ba (51). Full procedural blanks were <1% for all elements analyzed in this study, except for low concentration sample P9 where the Pb blank was 1.2%. Therefore, no blank correction was applied. Analytical precision and accuracy were determined using unleached United States Geological Survey (USGS) rock reference material BCR-2 (*n* = 22) and NIST SRM 2702 (*n* = 6) and repeat analyses of sample K8 (PS75/056-1, 52 to 53 cm; *n* = 12). The average 2RSD precision of the repeat analysis of BCR-2, NIST SRM 2702, and K8 was ∼5% for all elements analyzed during this study (*SI Appendix*, Table S1). The accuracy of BCR-2 and NIST SRM 2702 rock reference materials was better than 6.4% for all elements compared to reported reference values (*SI Appendix*, Table S1).

### Radiogenic Isotope Analyses.

The Pb, Nd, and Sr isotope compositions were determined using a Thermo Scientific *Neptune Plus* multicollector ICP-MS instrument at the Institute for Chemistry and Biology of the Marine Environment (ICBM) in Oldenburg. All reported AGV-1 and BCR-2 rock reference material results were obtained on leached residues (50, 51). For Nd isotope analyses, mass bias was corrected for using ^146^Nd/^144^Nd = 0.7219 and an exponential law. Isobaric interferences of ^142^Ce and ^144^Sm on ^142^Nd and ^144^Nd were monitored and corrected for by using ^140^Ce and ^147^Sm, respectively. The JNdi-1 reference material was measured every four samples to correct for the instrumental offset of the mass bias–corrected ^143^Nd/^144^Nd ratios of the samples to JNdi-1 reference ratio of 0.512115 ± 0.000007 (92). The external reproducibility (2SD) of normalized Nd isotope ratios of acid-leached BCR-2 and AGV-1 reference materials was 0.512643 ± 0.000014 (*n* = 36) and 0.512796 ± 0.000013 (*n* = 36), respectively. These results are in excellent agreement with literature ^143^Nd/^144^Nd of 0.512637 ± 0.000013 (93) for acid-leached BCR-2 residues and 0.512791 ± 0.000013 for unleached AGV-1 rock powder (94). Repeated analyses of samples C7 (^143^Nd/^144^Nd = 0.512387 ± 0.000011) (51) and K8 yielded ^143^Nd/^144^Nd = 0.512394 ± 0.000003 (2SD, *n* = 4) and ^143^Nd/^144^Nd = 0.512391 ± 0.000011 (2SD, *n* = 16), respectively. All sample Nd isotope results are expressed in epsilon notation, namely, as ε_Nd_ = (^143^Nd/^144^Nd_sample_)/(^143^Nd/^144^Nd_CHUR_) − 1] × 10^4^ where CHUR is the Chondritic Uniform Reservoir (95).

For Sr isotope analyses, mass bias was corrected for by using ^86^Sr/^88^Sr = 0.1194 and an exponential law (96). Krypton gas blanks, measured on ^83^Kr to correct for ^86^Kr on ^86^Sr, were below 0.1 mV, while ^86^Sr was measured at 2 to 3 V. Isobaric interferences from ^87^Rb on ^87^Sr were monitored and corrected using ^85^Rb. Repeat analyses of NIST SRM987 (every 4 samples) were used to normalize sample ^87^Sr/^86^Sr to SRM987 ^87^Sr/^86^Sr of 0.710248 (96). The external reproducibility of acid-leached BCR-2 and AGV-1 was ^87^Sr/^86^Sr = 0.704996 ± 0.000025 (2SD, *n* = 36) and 0.703955 ± 0.000023 (2SD, *n* = 36), respectively. These values are indistinguishable from the values reported previously for acid-leached residues of BCR-2 (0.705000 ± 0.000011) (93) and AGV-1 reference materials (0.703950 ± 0.000012) (94). Repeat analyses of samples C7 (Sr/^86^Sr = 0.710887 ± 0.000021) (51) and K8 yielded Sr/^86^Sr = 0.710882 ± 0.000022 (2SD, *n* = 5) and Sr/^86^Sr = 0.711354 ± 0.000017 (2SD, *n* = 20), respectively.

Measurements of the Pb isotope compositions were performed as standard-sample bracketing using NIST SRM981 as the bracketing standard (97). Isobaric interference of ^204^Hg on ^204^Pb was corrected for through monitoring ^202^Hg (<0.5 mV), while ^204^Pb was typically measured at ∼300 to 400 mV. The instrumental mass bias of sample analyses was corrected for by normalization of the measured SRM981 values to the accepted values of ref. 98. The external reproducibility (2SD) was monitored through repeat analyses of secondary rock reference materials BCR-2 and AGV-1. Selected Pb isotope results for BCR-2 (*n* = 45) were ^206^Pb/^204^Pb = 18.8015 ± 0.0026 (18.8029 ± 0.0010) (93), ^207^Pb/^204^Pb = 15.6250 ± 0.0020 (15.6239 ± 0.0008) (93), and ^208^Pb/^204^Pb = 38.8310 ± 0.0067 (38.8287 ± 0.0025) (93). Repeat analyses of AGV-1 (*n* = 45) yielded ^206^Pb/^204^Pb = 18.9025 ± 0.0037 (18.9054 ± 0.0013) (94), ^207^Pb/^204^Pb = 15.6148 ± 0.0017 (15.6165 ± 0.0001) (94), and ^208^Pb/^204^Pb = 38.5732 ± 0.0088 (38.5875 ± 0.0220) (94) for the same analytical sessions. It is noted that our results on acid-leached BCR-2 and AGV-1 rock reference materials are compared to the acid-leached literature results where available and that the reference ratios of acid-leached AGV-1 residues are based on only two analyses (94). Overall, our Pb isotope results on leached rock reference materials BCR-2 and AGV-1 demonstrate the excellent reproducibility of existing literature values (93, 94) and previous results from the ICBM laboratory (50, 51). Repeat analyses of sample K8 (*n* = 7) yielded ^206^Pb/^204^Pb = 18.9945 ± 0.0014, ^207^Pb/^204^Pb = 15.6575 ± 0.0011, and ^208^Pb/^204^Pb = 38.9859 ± 0.0034. Results of repeat measurements of sample R14 (*n* = 3) are ^206^Pb/^204^Pb = 18.9298 ± 0.0008, ^207^Pb/^204^Pb = 15.6528 ± 0.0004, and ^208^Pb/^204^Pb = 38.9092 ± 0.0016. We report the results for the reference material with the highest 2SD as 2SD uncertainty for all samples.

The procedural blanks for Nd, Pb, and Sr were below 60 pg, 0.8 ng, and 0.8 ng (one Sr blank with 1.6 ng), respectively. Typically, the blank contaminations were significantly less than 1% of the individual sample Nd, Pb, and Sr yields with negligible effects on the respective sample isotope compositions. For a subset of three samples with a Pb blank contamination of up to 3.3%, we applied a correction by using measured blank Pb isotope compositions (*SI Appendix* contains more details).

### Isotope Mixture Modeling.

The geochemical data were investigated using the Bayesian mixing model MixSIAR (99, 100), configured for detailed quantification of source contributions to individual samples (52). The model provides comprehensive mass balance constraints by including the compositional range of the endmembers and multiple isotope ratios (52, 99, 100). This is an improvement compared to previous dust provenance work using simple mass balance calculations with only two isotope ratios (26, 33, 34, 45, 46, 50, 51, 64, 65). The uncertainty of the model output is predominantly driven by the isotopic variability of the endmembers included in the model (*SI Appendix*, Table S2), and the 2SE of individual source contributions is typically <±0.5%. Due to the three different isotope systems used in this study, we include previously published Nd, Sr, and Pb concentration data (26, 51, 65) for the dust endmembers used in our model setup (*SI Appendix*, Table S2). All endmembers used for the modeling were defined based on their reported significance as dust sources in the Southern Hemisphere and their geochemical differences following previous approaches (51, 52). As a result, South American source regions are grouped by latitude (Fig. 1) (51). We revised the previously used endmember value for Southern Africa based on a more comprehensive dataset from Namibia (65) (*SI Appendix*, Table S2 contains more details). Western Australia was excluded as a potential source as the contributions from this region were below 2% throughout and therefore negligible (52). We use 1SD of source region isotope variability yielding smaller confidence intervals in the final output. Thus, we improve the differentiation between the contributions from individual source areas by excluding atypical compositions from individual source regions (52), which are unlikely to significantly influence the well-mixed signal of far-traveled mineral dust at the remote location of core PS75/056-1 (48). We do not consider Antarctic source regions as possible input because the dust fluxes reconstructed for PS75/056-1 are two orders of magnitude higher than dust fluxes reported for the Taylor Glacier near the main Antarctic dust sources (101).

## Supplementary Material

Supplementary File

Supplementary File

Supplementary File

## Data Availability

All data presented in this paper are included in this published article, its *SI Appendix* file, and Datasets S1 and S2. Datasets S1 and S2 are also available in the PANGAEA database under https://doi.org/10.1594/PANGAEA.947018 (106) and https://doi.org/10.1594/PANGAEA.947019 (107), respectively.
