# Supplementary material for: Systematic changes in circumpolar dust transport to the Subantarctic Pacific Ocean over the last two glacial cycles
Source: Proc Natl Acad Sci U S A. 2022 Nov 21;119(47):e2206085119. doi: 10.1073/pnas.2206085119 (PMC9704702; doi:10.1073/pnas.2206085119)
Supplement: Supplementary File [file pnas.2206085119.sapp.pdf]

PNAS
www.pnas.org

Supplementary Information for

Systematic changes in circumpolar dust transport to the Subantarctic Pacific Ocean during the last two glacial cycles

Torben Struve, Jack Longman, Martin Zander, Frank Lamy, Gisela Winckler and Katharina Pahnke

Corresponding author: t.struve@icbm.de

This PDF file includes:

- Supplementary texts 1 and 2
- Figures S1 to S6
- Tables S1 to S2
- Legend for Datasets S1 and S2
- SI References

Other supplementary materials for this manuscript include the following:

Dataset S1 and Dataset S2

Supplementary Information Text

Supplementary text 1: replicate sample compositions and blank levels.

Our dataset contains a total of 13 full replicate samples showing overall good reproducibility (Fig. S1). All replicate Nd isotope results fall within the applied 2SD uncertainty range (Fig. S1A) (see also Methods section of the main text). However, some replicate samples show moderate $^{87}Sr/^{86}Sr$ offsets (significant at the applied 2SD level, i.e. ±0.000026) (Fig. S1B), despite low Sr blank levels of <1% (see Methods section of the main text). This may indicate sample heterogeneity. Yet, the total range of Sr isotope changes in our downcore dataset is one order of magnitude larger ($\Delta^{87}Sr/^{86}Sr$ of 0.0057) than the maximum Sr isotope offset in our replicate data ($\Delta^{87}Sr/^{86}Sr$ <0.00044 with the exception of sample P6 (62–63 cm depth) where $\Delta^{87}Sr/^{86}Sr$ = 0.001; see Dataset S1). Moreover, the maximum Sr isotope offsets are about two orders of magnitude smaller than the compositional range of the Southern Hemisphere potential dust source areas (see Table S2). Therefore, the observed Sr isotope deviations between our replicate samples are considered negligible even though they exceed our calculated 2SD uncertainty range.

For Pb isotopes, we find n = 7 replicates to be within the applied 2SD uncertainty range of ±0.0037 for $^{206}Pb/^{204}Pb$ (based on replicate analyses of rock reference material AGV-1; see Methods section of the main text for details) (Fig. S1C and Dataset S1). For sample batches M, N and O we observed elevated Pb blank levels of up to 0.82 ng (n = 6 blanks) as compared to an average of 0.18 ng for the remaining six sample batches (n = 12 blanks). The combination of low lithogenic content and elevated blank levels yielded Pb blank contributions above 1% of the total Pb in 10 samples of our dataset, seven of which fall in the range of 1.0 to 1.6 %. The Pb blank contributions to replicate samples O5 and P13 (657–658 cm depth) are 1.6 and 0.4%, respectively. Yet, their Pb isotope values are identical suggesting that the Pb isotope compositions of our samples are largely unaffected by blank levels up to 1.6% introduced during sample processing in our labs. The similarity of our sample Pb/La = 0.4 ± 0.2 (2SD, n = 108) with the average upper continental crust (UCC) value of 0.67 (1) and a good correlation of Pb concentrations with lithogenic elements such as Zr (R^2 = 0.74, n = 108) or Nd (R^2 = 0.75, n = 108) exclude a significant contaminant Pb component in our samples. Only samples O11 and P14 from 707-708 cm depth show Pb/La ratios of ~0.8 (Dataset S1), i.e. slightly elevated above the UCC value of 0.67 (1). However, the identical Pb isotope compositions of these replicate samples (processed in different sample batches with different blank levels; see above) suggest that their different Pb/La reflects a change in dust composition/provenance rather than Pb contamination. The highest blank contributions of 2.1, 2.9 and 3.3% are found in samples M12 (397–398 cm depth), M13 (417–418 cm depth) and M14 (432–433 cm depth), respectively. For these three samples we use the corrected Pb isotope values, which leads to a reduction in the $^{206}Pb/^{204}Pb$ offset between sample M13 and duplicate sample P9 from 0.03 to 0.009, i.e. similar to the other three replicates that show $\Delta^{206}Pb/^{204}Pb$ slightly outside the applied 2SD uncertainty range (see Fig. S1C). Notably, the changes in source apportionment resulting from these corrections are small (<3.4% for continental-scale PSA) (see also Dataset S2).

Replicate samples N5 and P11 (477–478 cm depth) show a $\Delta^{206}Pb/^{204}Pb$ of 0.034 (Fig. 1C), which is, to our best knowledge, unrelated to sample contamination with blank levels contributing less than 0.2% to both samples. Similarly, the largest $^{206}Pb/^{204}Pb$ offset at 62–63 cm depth (between sample P6 and samples K10 and R14 with $\Delta^{206}Pb/^{204}Pb$ of 0.048 and 0.051, respectively) cannot be ascribed to sample contamination as blank levels are below 0.3% for all three samples. In both cases, the more radiogenic $^{206}Pb/^{204}Pb$ correspond with less radiogenic $^{87}Sr/^{86}Sr$ (samples N5 and P6) relative to samples P11 and K10/R14 from the same depths (Fig. 1B, C). Considering this correspondence between these two independent parameters, we ascribe the offsets at these two depths to sample heterogeneity.

Importantly, the largest isotopic offset between samples R14 and P6 at 62-63 cm depth results in a moderate change of ~5% for continental-scale dust source quantification, which has a negligible influence on the dust provenance time series from core PS75/056-1.

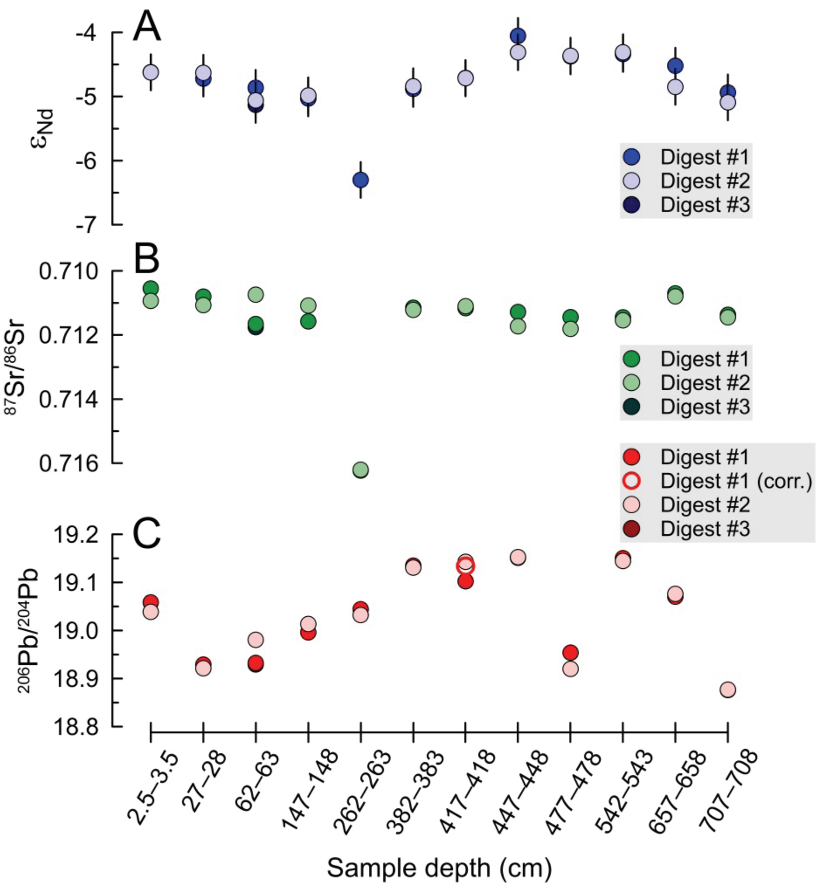

Fig. S1. Radiogenic isotope results for dust fraction replicate samples of core PS75/056-1. (A) Neodymium (Nd) isotope data, (B) strontium (Sr) isotope data and (C) lead (Pb) isotope data ($^{206}Pb/^{204}Pb$ depicted). Error bars show the 2SD uncertainty (see Methods of the main text for more details) where the 2SD is larger than the symbol size. 'Digest' refers to the individual sample digests. All sample results plotted against depth below seafloor. No Nd isotope data available for the replicate sample at 262–263 cm depth. Note that pale symbols (digest #2) may overlie other symbols where the isotopic compositions are similar. Blank correction applied to sample digest #1 at 417-418 cm depth ('corr.') due to elevated blank contribution in this sample (see Supplementary text 1 for details).

Supplementary text 2: rare earth element characteristics.

Several samples from the top ~90 cm of core PS75/056-1 show a positive Ce anomaly (Ce/Ce* = 3 × Ce_N/[2 × La_N + Pr_N], subscript 'N' indicates PAAS-normalized values (1)) of up to 1.6 reflected in the large 2SD of the PAAS and Yb-normalized Ce results in Fig. S2A. Notably, Ce(III), unlike the other REE (and Pb, Sr), can be oxidized to a solid Ce(IV) state in the marine environment resulting in more efficient removal of Ce from aqueous solution and Ce enrichment in particular in hydrogenetic ferromanganese (oxy)hydroxide precipitates (2, 3) (Fig. S2A). Therefore, the positive Ce anomaly may indicate incomplete removal of hydrogenetic Ce carrier phases from these samples, for example related to an increased presence of hydrogenetic metal precipitates and/or re-precipitation of Ce at the end of the sequential leaching procedure. Incomplete removal of hydrogenetic Ce would be consistent with a positive correlation between Ce/Ce* and Ce concentrations in the sediment samples from the upper 90 cm of the core (R^2 of 0.52, n = 24). However, the concentrations of Pb, Sr and the other REEs as well as the $HREE_N/LREE_N$ ratios (excluding Ce) show no significant correlation with the Ce anomaly in this core section (e.g., R^2 of 0.12 for Ce/Ce* and Nd concentrations). There is also no significant correlation between the magnitude of the Ce anomaly and the Pb, Nd or Sr isotope compositions. Thus, the overall flat pattern of our shale-normalized REE data (Fig. S2A) and their similarity with previously published <5 µm fraction results from terrestrial source regions and South Pacific marine sediments (Fig. S2B) suggest a crustal origin for the Nd, Sr and Pb isotope tracer signals used for the reconstruction of dust provenance changes in this study.

Table S1: Reproducibility of trace element analysis of rock reference materials used in this study. Reference values for NIST SRM 2702 represent certified values (normal font) and information values (in brackets) with 95% confidence level (CL) as reported in the NIST SRM 2702 certificate of analysis. All concentration data in µg/g. Reference values for BCR-2 from ref. (4).

	Sr	Zr	Ba	La	Ce	Pr	Nd	Sm	Eu	Gd	Tb	Dy	Ho	Er	Tm	Yb	Lu	Pb
**Average BCR-2 (n=22)**	**329.2**	**174.5**	**672.8**	**26.3**	**56.5**	**7.2**	**29.7**	**6.9**	**1.93**	**6.7**	**1.1**	**6.8**	**1.37**	**3.79**	**0.52**	**3.53**	**0.50**	**11.0**
2SD	22.6	25.3	9.0	1.4	3.1	0.3	1.0	0.4	0.1	0.4	0.1	0.3	0.08	0.20	0.03	0.24	0.04	1.0
2RSD (%)	6.9	14.5	1.3	5.3	5.5	4.2	3.2	5.8	5.6	6.6	5.8	4.8	5.56	5.18	6.60	6.68	8.22	9.5
**Reference values**	**337.4**	**186.5**	**683.9**	**25.1**	**53.1**	**6.8**	**28.3**	**6.5**	**2.0**	**6.8**	**1.1**	**6.4**	**1.3**	**3.7**	**0.5**	**3.4**	**0.5**	**10.6**
CL (95%)	2.0	0.8	0.7	0.6	0.6	0.6	1.3	0.7	1.2	1.1	2.4	0.9	0.8	1.0	1.1	1.1	1.5	1.6
Offset to ref. val. (%)	-2.4	-6.4	-1.6	4.9	6.4	4.8	5.0	6.1	-3.1	-1.1	6.4	6.3	4.2	3.3	-1.9	4.1	-0.8	3.7
**Average NIST2702 (n=6)**	**118.9**	**335.7**	**381.0**	**68.9**	**120.5**	**15.9**	**60.0**	**11.2**	**2.0**	**9.4**	**1.4**	**7.9**	**1.55**	**4.23**	**0.59**	**3.96**	**0.57**	**135.6**
2SD	1.8	3.9	5.8	1.0	2.0	0.3	1.6	0.4	0.0	0.3	0.0	0.3	0.1	0.1	0.0	0.2	0.0	7.0
2RSD (%)	1.5	1.2	1.5	1.5	1.7	1.9	2.7	3.8	2.3	3.4	3.1	3.9	3.7	2.9	4.1	5.2	4.6	5.2
**Reference values**	**119.7**	**–**	**397.4**	**73.5**	**123.4**	**–**	**(56)**	**(10.8)**	**–**	**–**	**–**	**–**	**–**	**–**	**–**	**–**	**–**	**132.8**
CL (95%)	3	–	3.2	4.2	5.8	–	–	–	–	–	–	–	–	–	–	–	–	1.1
Offset to ref. val. (%)	-0.7	–	-4.1	-6.3	-2.4	–	7.1	4.2	–	–	–	–	–	–	–	–	–	2.1
**Average K8 (n=12)**	**117.4**	**104.1**	**451.7**	**12.2**	**32.5**	**2.9**	**10.6**	**2.2**	**0.46**	**2.03**	**0.32**	**1.94**	**0.40**	**1.16**	**0.17**	**1.21**	**0.17**	**5.01**
2SD	4.0	4.0	3.3	0.3	1.4	0.1	0.4	0.1	0.03	0.10	0.02	0.08	0.02	0.05	0.01	0.09	0.01	0.22
2RSD (%)	3.4	3.8	0.7	2.6	4.4	4.0	3.4	6.2	5.8	5.1	4.9	4.1	5.1	4.6	6.2	7.7	8.3	4.5

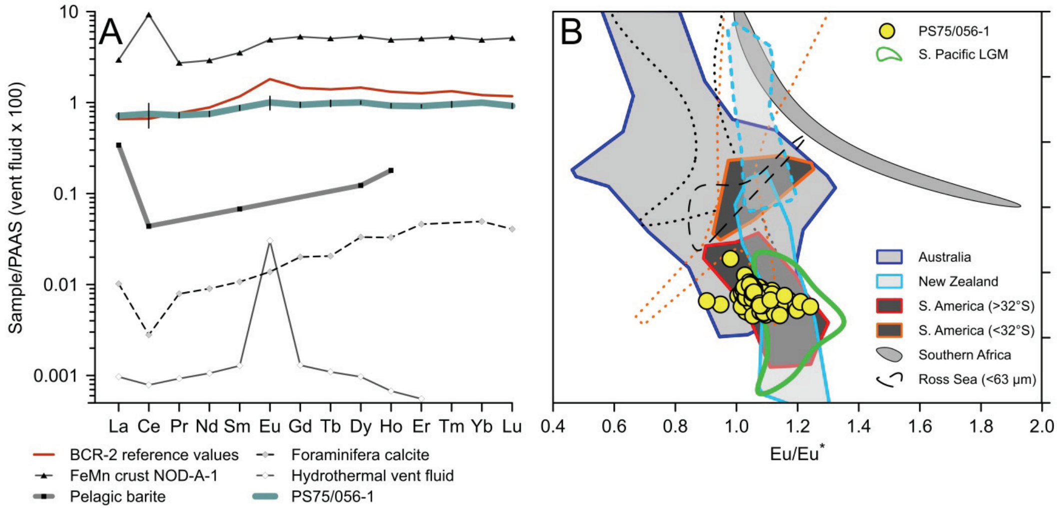

Fig. S2. Rare earth element results of PS75/056-1 dust fraction samples. (A) Fine fraction rare earth element (REE) data normalized to Post-Archean Australian Shale (PAAS) (1) and to Yb, shown as averages and their 2SD (n = 108, black error bars). The results are compared with USGS rock reference material BCR-2 (4), hydrothermal vent fluids (5) and important trace metal rich authigenic phases in marine sediments including hydrogenetic ferromanganese precipitates (USGS reference material NOD-A-1) (3, 6), pelagic barite (physically separated microcrystals, sample Proa 101) (7) and foraminifera calcite (cleaned coretop *G. sacculifer* from Southeast Pacific core 54MC) (8). (B) PS75/056-1 fine fraction REE results illustrated as shale-normalized (subscript `N´) La/Yb ratio versus europium anomaly ($Eu/Eu^* = 3 \times Eu_N/[2 \times Sm_N + Gd_N]$). South Pacific LGM comprises open ocean locations from the time period 18–24 ka BP (9). Published potential source area (PSA) data from South America (<5 µm) (10), New Zealand (dashed outline: bulk loess, glacial dust, dust traps) (11) (solid outline: <5 µm fraction) (12), East Australia (river sediments of <25 µm, <80 µm, <90 µm size fractions, and bulk sediment) (11, 13, 14, 15), Southern Africa (<5 µm) (16) and the LGM mid-latitude South Pacific (<5 µm) (9). Note that the published dust fraction REE data may not represent the full range of Southern Hemisphere PSA compositions. For example, South America (<32°S) comprises six <5 µm fraction samples plotting in a confined space in (B), whereas the <63 µm fraction from the same region comprises 19 samples (10) which occupy a significantly larger space (stippled orange polygon). Similarly, available REE data exist for only 3 Southern African <5 µm fraction samples, but for 16 bulk sediment samples (16) indicating a much larger compositional range (stippled black polygon).

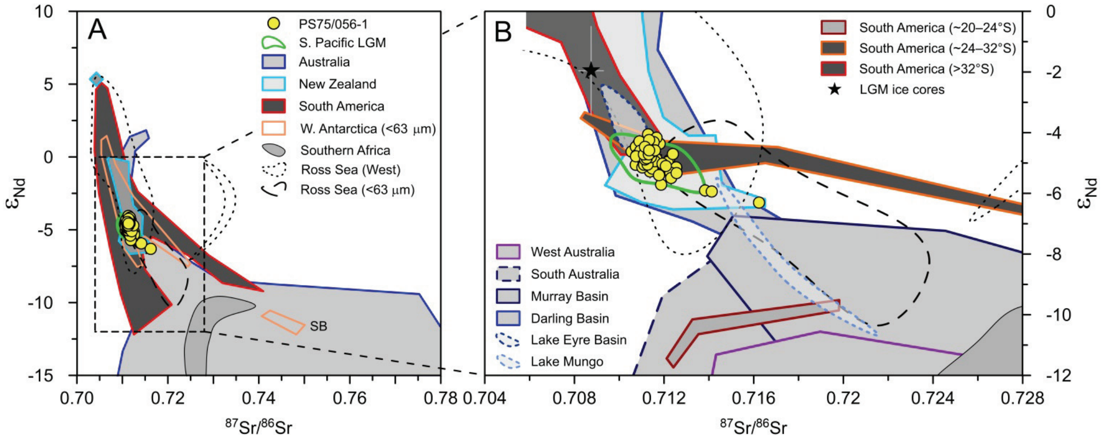

Fig. S3: Fine fraction samples of South Pacific core PS75/056-1 in Nd–Sr isotope space. Last Glacial Maximum (LGM) time slice samples from ref. (9). Ross Sea shelf data (<63 μm fraction) represent the compositional range of IRD exported into the South Pacific (9, 17, 18). Other potential source area (PSA) data as in Table S2. (A) Overview of Southern Hemisphere dust fraction compositions. SB: Sulzberger Bay. (B) Detailed view with South American and Australian sources grouped into sub-continental scale PSAs based on their geochemical composition (see main text for more details). LGM average values for Antarctic ice cores calculated using Dome B and C samples (ε_{Nd} = -2.0 ± 1.5, $^{87}Sr/^{86}Sr$ = 0.7087 ± 0.0006, 2SD, n = 9) (19, 20).

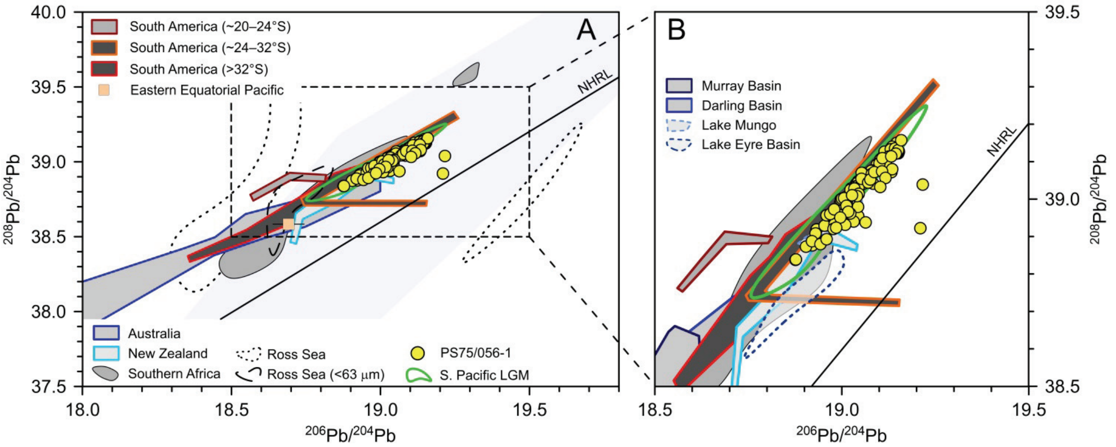

Fig. S4: Fine fraction samples of South Pacific core PS75/056-1 in Pb–Pb isotope space. Last Glacial Maximum (LGM) time slice samples from ref. (9). Other potential source area (PSA) data as listed in Table S2. (A) Overview of Southern Hemisphere dust fraction $^{206}Pb/^{204}Pb$. LGM average value for the Eastern Equatorial Pacific from ref. (21). Light blue shading indicates EDC ice core data (22). NHRL: Northern Hemisphere Reference Line (23). Ross Sea shelf data (<63 μm fraction) represent the compositional range of IRD exported into the South Pacific (9, 17, 18). (B) Detailed view with Australian sources grouped into sub-continental scale PSAs based on their geochemical composition (see main text for more details).

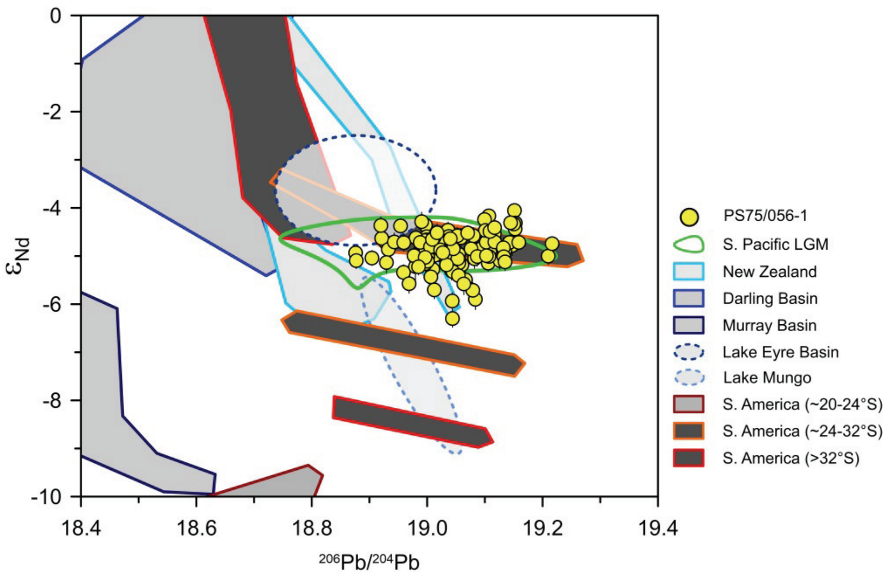

Fig. S5: Fine fraction samples of South Pacific core PS75/056-1 in Pb–Nd isotope space. Last Glacial Maximum (LGM) time slice samples from ref. (9). Other PSA data as in Table S2. Note that Southern Africa is not shown here, because the fine fraction samples included in our mixing model setup are characterized by unradiogenic Nd isotope compositions of ~-10 to ~-17 (16) (see Fig. S3).

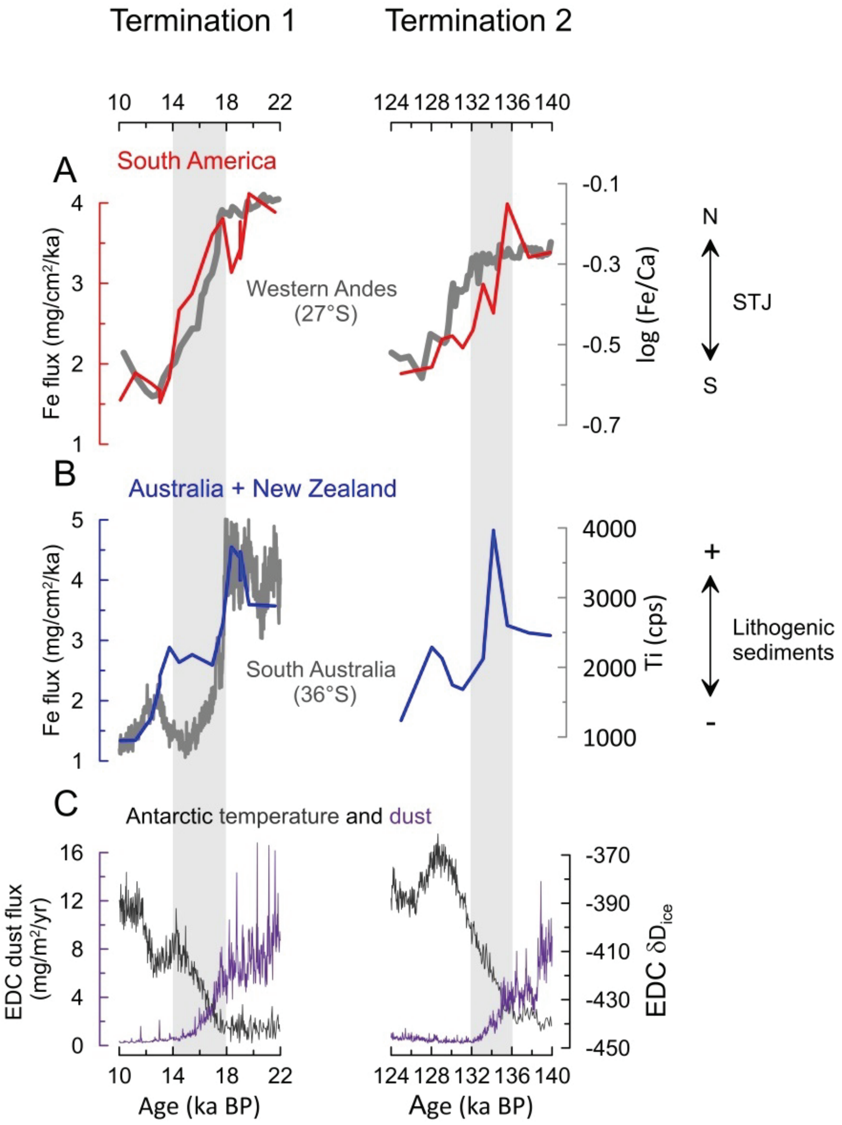

Fig. S6: Dust-Fe input changes in the Subantarctic South Pacific during terminations 1 and 2. (A) South American dust-Fe fluxes compared to the presence of the subtropical jetstream (STJ) at the latitude of main dust source areas in Central South America (24). (B) Dust-Fe fluxes from Australian and New Zealand sources compared to lithogenic sediment input at site MD03-2611 off South Australia (not available for termination 2) (25). (C) Antarctic ice core temperature (26) and dust flux changes for terminations 1 and 2 (27) as stratigraphic and climatic references, respectively. Grey bars indicate early deglacial intervals. Note that millennial-scale offsets between the different records may (partly) result from age model uncertainties (see also main text).

Table S2. Radiogenic isotope composition of potential dust source areas in the Southern Hemisphere used for the Bayesian modelling (28). Data are taken from the compilation of ref. (9) with revised values for Lake Eyre, Lake Mungo, New Zealand (12) and Southern Africa (16). AUS: Australia. NZ: New Zealand. SAM: South America. SAF: Southern Africa.

	A posteriori group	$^{206}Pb/^{204}Pb$	1SD	$^{207}Pb/^{204}Pb$	1SD	$^{208}Pb/^{204}Pb$	1SD	n	ε_{Nd}	1SD	$^{87}Sr/^{86}Sr$	1SD	n
**Lake Eyre Basin**	AUS+NZ	18.89	0.08	15.64	0.01	38.74	0.08	9	-3.6	0.6	0.7103	0.0005	9
**Murray Basin**	AUS+NZ	18.48	0.10	15.62	0.01	38.50	0.10	8	-9.1	1.7	0.7316	0.017	24
**Darling Basin**	AUS+NZ	18.51	0.28	15.61	0.03	38.49	0.31	11	-2.6	2.1	0.7108	0.001	19
**West Australia**[+]	–	17.95	0.03	15.61	0.01	38.02	0.13	3	-17.0	4.2	0.7381	0.023	21
**Lake Mungo**	AUS+NZ	18.97	0.10	15.65	0.02	38.94	0.08	3	-8.4	2.6	0.7178	0.004	3
**New Zealand**	AUS+NZ	18.84	0.08	15.66	0.01	38.76	0.09	20	-4.9	1.9	0.7124	0.002	20
**S. America 20-24°S**	SAM	18.69	0.11	15.67	0.01	38.85	0.08	3	-10.5	0.9	0.7150	0.004	3
**S. America 24-32°S (endmember)**[§]	SAM	19.25	0.001	15.68	0.001	39.31	0.003	1	-5.0	0.1	0.7169	0.00001	1
*S. America 24-32°S (total)**	–	*18.96*	*0.20*	*15.66*	*0.02*	*38.90*	*0.22*	*6*	*-4.7*	*1.0*	*0.7160*	*0.0063*	*6*
**S. America >32°S**	SAM	18.70	0.15	15.64	0.01	38.65	0.16	21	-1.3	3.4	0.7102	0.007	27
**Namibia**[#]	SAF	18.94	0.31	15.72	0.04	38.99	0.52	14	-13.0	2.7	0.7277	0.004	18

[§]Endmember composition as defined by ref. (9).

#Values calculated from fine fraction results for Huab, and Sea sand regions, and Omaruru bulk results (16).

*Average of source data for this region (10, 29) included here for completeness.

[+]Excluded from the final endmember modelling due to overall very low (<2%) and largely invariable contributions (28).

Dataset S1. Geochemical results for <5 μm size fraction sediments of core PS75/056-1. Sediment core PS75/056-1 was recovered from 3581.0 m water depth at -55.162°N/-114.789°E during expedition ANT-XXVI/2 (PS75 BIPOMAC) in 11/2009-01/2010. Full replicate samples in Italics. Age model from ref. (1). See main text and SI Appendix for further details.

Dataset S2. Source apportionment results for <5 μm size fraction sediments of core PS75/056-1. Mean values of dust grain size taken from ref. (1). Iron (Fe) and Fe(II) flux data from ref. (2) were interpolated linearly to obtain values for sample intervals used for dust provenance reconstructions. The source-specific Fe fluxes were calculated based on the relative contributions of the individual dust source regions to the total Fe input. All data are shown on the age model of ref. (1). See main text for further details on source apportionment modelling.

SI References

1. S. R. Taylor, S. M. McLennan, The continental crust: Its composition and evolution. Blackwell Scientific Publications (1985), 312 pp.
2. H. J. W. de Baar, K. W. Bruland, J. Schijf, S. M. A. C. van Heuven, M. K. Behrens, Low cerium among the dissolved rare earth elements in the central North Pacific Ocean. *Geochim. Cosmochim. Acta* **236**, 5–40 (2018).
3. M. Bau, A. Koschinsky, Oxidative scavenging of cerium on hydrous Fe oxide: Evidence from the distribution of rare earth elements and yttrium between Fe oxides and Mn oxides in hydrogenetic ferromanganese crusts. *Geochem. J.* **43**, 37–47 (2009).
4. K. P. Jochum, *et al.*, Reference Values Following ISO Guidelines for Frequently Requested Rock Reference Materials. *Geostand. Geoanal. Res.* **40**, 333–350 (2016).
5. G. P. Klinkhammer, H. Elderfield, J. M. Edmond, A. Mitra, Geochemical implications of rare earth element patterns in hydrothermal fluids from mid-ocean ridges. *Geochim. Cosmochim. Acta* **58**, 5105–5113 (1994).
6. P. Dulski, Reference Materials for Geochemical Studies: New Analytical Data by ICP-MS and Critical Discussion of Reference Values. *Geostand. Newsl.* **25**, 87–125 (2001).
7. F. Guichard, T. M. Church, M. Treuil, H. Jaffrezic, Rare earths in barites: distribution and effects on aqueous partitioning. *Geochim. Cosmochim. Acta* **43**, 983–997 (1979).
8. B. A. Haley, G. P. Klinkhammer, A. C. Mix, Revisiting the rare earth elements in foraminiferal tests. *Earth Planet. Sci. Lett.* **239**, 79–97 (2005).
9. T. Struve, *et al.*, A circumpolar dust conveyor in the glacial Southern Ocean. *Nat. Commun.* **11**, 5655 (2020).
10. S. Gili, *et al.*, Glacial/interglacial changes of Southern Hemisphere wind circulation from the geochemistry of South American dust. *Earth Planet. Sci. Lett.* **469**, 98–109 (2017).
11. S. K. Marx, B. S. Kamber, H. A. McGowan, Provenance of long-travelled dust determined with ultra-trace-element composition: a pilot study with samples from New Zealand glaciers. *Earth Surf. Process. Landf.* **30**, 699–716 (2005).
12. B. G. Koffman, *et al.*, New Zealand as a source of mineral dust to the atmosphere and ocean. *Quat. Sci. Rev.* **251**, 106659 (2021).
13. G. B. Douglas, C. M. Gray, B. T. Hart, R. Beckett, A strontium isotopic investigation of the origin of suspended particulate matter (SPM) in the Murray-Darling River system, Australia. *Geochim. Cosmochim. Acta* **59**, 3799–3815 (1995).

14. S. K. Marx, B. S. Kamber, Trace-element systematics of sediments in the Murray–Darling Basin, Australia: Sediment provenance and palaeoclimate implications of fine scale chemical heterogeneity. *Appl. Geochem.* **25**, 1221–1237 (2010).

15. H. A. McGowan, B. Kamber, G. H. McTainsh, S. K. Marx, High resolution provenancing of long travelled dust deposited on the Southern Alps, New Zealand. *Geomorphology* **69**, 208–221 (2005).

16. S. Gili, *et al.*, South African dust contribution to the high southern latitudes and East Antarctica during interglacial stages. *Commun. Earth Environ.* **3**, 1–12 (2022).

17. G. Lang Farmer, K. Licht, R. J. Swope, J. Andrews, Isotopic constraints on the provenance of fine-grained sediment in LGM tills from the Ross Embayment, Antarctica. *Earth Planet. Sci. Lett.* **249**, 90–107 (2006).

18. S. R. Hemming, *et al.*, Strontium isotope tracing of terrigenous sediment dispersal in the Antarctic Circumpolar Current: Implications for constraining frontal positions. *Geochem. Geophys. Geosyst.* **8**, Q06N13 (2007).

19. B. Delmonte, *et al.*, Comparing the Epica and Vostok dust records during the last 220,000 years: stratigraphical correlation and provenance in glacial periods. *Earth-Sci. Rev.* **66**, 63–87 (2004).

20. B. Delmonte, *et al.*, Causes of dust size variability in central East Antarctica (Dome B): Atmospheric transport from expanded South American sources during Marine Isotope Stage 2. *Quat. Sci. Rev.* **168**, 55–68 (2017).

21. S. Pichat, W. Abouchami, S. J. G. Galer, Lead isotopes in the Eastern Equatorial Pacific record Quaternary migration of the South Westerlies. *Earth Planet. Sci. Lett.* **388**, 293–305 (2014).

22. P. Vallelonga, *et al.*, Lead isotopic compositions in the EPICA Dome C ice core and Southern Hemisphere Potential Source Areas. *Quat. Sci. Rev.* **29**, 247–255 (2010).

23. S. R. Hart, A large-scale isotope anomaly in the Southern Hemisphere mantle. *Nature* **309**, 753–757 (1984).

24. F. Lamy, *et al.*, Precession modulation of the South Pacific westerly wind belt over the past million years. *Proc. Natl. Acad. Sci. U.S.A.* **116**, 23455–23460 (2019).

25. P. De Deckker, M. Moros, K. Perner, E. Jansen, Influence of the tropics and southern westerlies on glacial interhemispheric asymmetry. *Nat. Geosci.* **5**, 266–269 (2012).

26. J. Jouzel, *et al.*, Orbital and Millennial Antarctic Climate Variability over the Past 800,000 Years. *Science* **317**, 793–796 (2007).

27. F. Lambert, M. Bigler, J. P. Steffensen, M. Hutterli, H. Fischer, Centennial mineral dust variability in high-resolution ice core data from Dome C, Antarctica. *Clim. Past* **8**, 609–623 (2012).

28. J. Longman, T. Struve, K. Pahnke, Spatial and Temporal Trends in Mineral Dust Provenance in the South Pacific—Evidence From Mixing Models. *Paleoceanogr. Paleoclimatol.* **37**, e2021PA004356 (2022).

29. S. Gili, *et al.*, Provenance of dust to Antarctica: A lead isotopic perspective. *Geophys. Res. Lett.* **43**, 2291–2298 (2016).
